# Localized AL amyloidosis: A suicidal neoplasm?

**DOI:** 10.3109/03009734.2012.654861

**Published:** 2012-04-19

**Authors:** Per Westermark

**Affiliations:** Department of Immunology, Genetics and Pathology, Uppsala University, SE 751 85 Uppsala, Sweden

**Keywords:** Amyloid, immunoglobulin light chain, fibrils, giant cells, plasmacytoma, neoplasm

## Abstract

Although AL amyloidosis usually is a systemic disease, strictly localized AL deposits are not exceptionally rare. Such case reports form a considerable body of published articles. Although both AL amyloidosis types are formed from an N-terminal segment of a monoclonal immunoglobulin light chain, a typical localized AL amyloid differs from the systemic counterpart by the morphological appearance of the amyloid, and presence of clonal plasma cells and of giant cells. In this article it is pointed out that localized AL amyloidosis (‘amyloidoma') represents a true plasma cell neoplasm and not a pseudotumor. The pathogenesis of localized AL amyloidosis may differ from that of the systemic type, a suggestion underlined by the fact that localized AL amyloidosis of kappa type is as common as that of lambda origin, in contrast to the systemic form where lambda chains constitute the overwhelming majority of cases. It is suggested that oligomeric assemblies of the produced immunoglobulin light chain are toxic to plasma cells, which in this way commit suicide.

## Introduction

The amyloidoses constitute a large and heterogeneous group of protein misfolding disorders in which proteins adopt a high degree of beta-sheet structure, allowing molecules to bind to each other in long, cross-beta-sheet fibrils. In this form the protein is comparatively resistant to degradation, explaining why the amyloid masses may increase as long as the protein substrate is present. The mechanisms behind the fibril formations are still insufficiently understood, but it is generally accepted that generation of amyloid from a protein includes three phases. The first phase is nucleation, at which misfolded molecules assemble to form a nucleus, resembling the process of crystallization (1). This nucleus can catalyze an amyloid-prone misfolding of other molecules, which adopt β-structure and form rapidly growing fibrils. This second elongation phase proceeds until a steady state (third phase) has been obtained. A particularly favorable milieu is consequently formed where there is a high concentration of substrate.

This mini-review deals with the nature of one localized form of amyloidosis that clinically often presents as a malignant tumor but that is often regarded as a pseudotumor or tumor-like nodule (2,3). One of the main messages in this communication is that it is a manifestation of a real neoplasm.

## AL amyloidosis

Amyloidosis can either be systemic or localized. In systemic forms, the precursor of the fibril protein is expressed at one site, e.g. liver or bone-marrow, released to blood plasma in soluble form, distributed via the circulation, and finally deposited as amyloid fibrils in different organs by yet unknown mechanisms. In human, 15 different proteins have been shown to be able to cause systemic amyloidosis, although most forms are very rare (4). One of the least uncommon is AL amyloidosis where the fibril protein is derived from monoclonal immunoglobulin (Ig) light chains. The primary cause of this form of amyloid is consequently a clonal expansion of one plasma cell expressing one light chain, usually in excess. The plasma cell clone is spread in the bone-marrow, either as a malignant tumor (myeloma) or as apparently benign cells. The Ig light chain circulates in blood plasma before it aggregates into amyloid fibrils in virtually all organs outside the brain by so far unknown mechanisms. Systemic AL amyloidosis is usually lethal.

Although many of the subclasses of Ig light chains are represented in amyloid only, not all light chains are amyloidogenic. There is strong evidence that this restriction depends on the variability in the amino acid sequences, and certain variable subgroups and amino acid substitutions are particularly important (5,6). AL protein of lambda type is 2–3 times more common than of kappa type (7,8). The major part of amyloid AL protein lacks a portion of the constant region (9-12), although a minor component always is the complete Ig light chain (13). Whether or not this truncation is pathogenically important or occurs after fibrillogenesis is not clear.

### Localized AL amyloidosis

**Figure 1.**
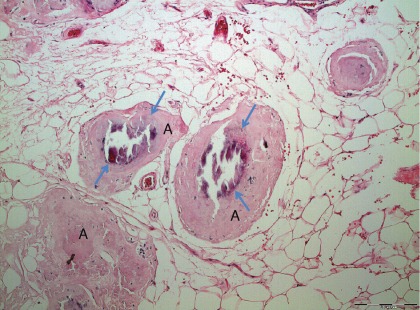
Localized AL amyloidosis in a breast. The localized nature and occurrence of microcalcifications (arrows) constitute a risk of misdiagnosis of mammary carcinoma.

Amyloid localized to one tissue, where multiple deposits appear, is very common, particularly in association with aging. Examples are amyloid in the brain in Alzheimer's disease and in islets of Langerhans in type 2 diabetes (14). In contrast localized AL amyloidosis usually appears as one, often tumor-like lesion, although multiple nodules may occur. The symptoms may vary depending on location of the lesion, but, initially, malignant tumors are often suspected, particularly when the nodules are present in the breast, lung, or urinary tract (3,15,16). Calcifications are common (17), and in the case of breast nodules mammographic appearance may include microcalcifications, increasing the suspicion of malignancy (Figure 1) (18,19). A monoclonal component in plasma is usually absent (20). Treatment of choice seems to be local resections endoscopically, although radical excision has been performed successfully, e.g. for respiratory lesions (3,21). Local resections often have to be repeated (22). The long-term prognosis is usually favorable, although deaths due to severe hemorrhages or other complications have been described (20,21). While a monoclonal gammopathy of unknown significance (MGUS) may develop to systemic amyloidosis or myeloma (23) this does not seem to happen with a localized plasma cell clone associated with localized amyloidosis, which almost never progresses to a systemic disease (20,24,25).

### Appearance of localized AL amyloid

**Figure 2.**
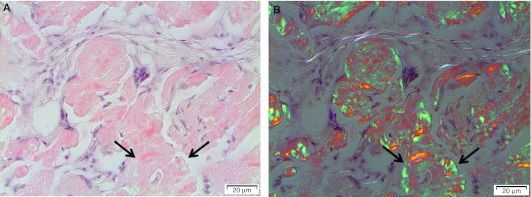
Typical appearance of localized AL amyloidosis with discrete amyloid formation and multiple giant cells. Note the concentric appearance of some amyloid particles (arrows).

Usually, amyloid deposits are observed in a strictly limited but not encapsulated area. Involvement of vessel walls is common but does only occur very close to the main deposit. Like systemic AL amyloidosis, affinity for Congo red is variable between patients but always present, as is the concomitant green birefringence in polarized light. Unlike deposits in most cases of systemic amyloidosis, crystal-like structures with Maltese cross appearance are as common as areas with concentric ring structures (Figure 2). These formations usually have stronger affinity for Congo red and a brighter green birefringence. Typically, scattered and small groups of plasma cells occur in the amyloid deposits. Localized AL amyloid can occur at virtually any site of the body, including the brain (26). Most common sites are eyelids, larynx, bronchi, skin, and urinary tract.

### Biochemical nature of localized AL amyloid

An immunoglobulin light chain nature of localized nodular pulmonary amyloid was indicated by peptide mapping and immunodiffusion soon after the monoclonal light chain origin of primary systemic amyloidosis had been demonstrated (27). Later, amino acid sequence analyses proved the monoclonal immunoglobulin light chain nature in a number of cases (28-35). AL kappa is as common as AL lambda (Solomon A., personal communication). On very rare occasions, a heavy chain fragment has been demonstrated (36). As in systemic AL amyloidosis, most of the amyloid protein is not full-length light chain molecules, and a varying part of the constant region is missing (27,29,34,35,37). Small amounts of full-length light chains are usually present as well as small C-terminal fragments (13). The importance of fragmentation in the amyloidogenic process is still not quite settled, since it has been suspected to occur after fibril formation. On the other hand, the almost universal predominance of N-terminal fragments over fragments from the C-terminus has been suggested to indicate a major mechanism in fibrillogenesis (37).

### Is localized AL amyloid a pseudotumor or a real neoplasm?

According to Celsus a tumor originally meant swelling, but the designation is nowadays almost only used for neoplasm. Consequently, tumor-like lesions should be non-neoplastic swellings. It is obvious that localized AL amyloidosis appears as a tumor according to a classical definition. The descriptions ‘tumor-like' or ‘pseudotumor' are often used in order to discriminate from neoplasm. This may be correct for localized AL amyloidosis in the sense that the mass depends on the deposition of amyloid, but, as described below, the basic lesion is nevertheless a neoplasm in the form of a clonal expansion of one plasma cell.

**Figure 3.**
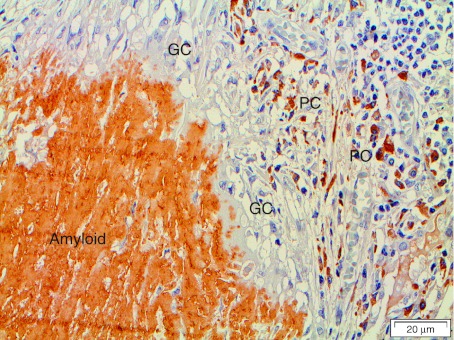
‘Amyloidoma' immunolabeled with the immunoglobulin light chain lambda-specific monoclonal antibody pwlam (73). Amyloid and a majority of plasma cells (PC) are strongly labeled. Note the giant cells (GC) between plasma cells and amyloid. The hypothesis is that plasma cells are synthesizing an amyloidogenic Ig light chain, which is modified by giant cells to amyloid fibrils.

A localized production of the amyloid immunoglobulin light chain was suspected at an early date but was difficult to prove. On most occasions, the plasma cell clone is very discrete and even difficult to recognize. Only very exceptionally is the background a clear-cut immunocytoma or plasmacytoma with atypia and mitoses (38). Not only is the number of plasma cells in the amyloid lesion often fairly sparse, but trials to identify monoclonality of the plasma cells by immunohistochemistry have also generally failed (22), although an abnormal overload of kappa or lambda plasma cells sometimes is evident (Figure 3). By means of immunohistochemistry it is often evident that a mixture of plasma cell populations with both kappa and lambda-expressing cells is present. A predominance of one plasma cell type (kappa or lambda) has sometimes been taken as evidence for monoclonality (39). However, gene rearrangement analysis of a few typical localized AL amyloidoses has shown presence of plasma cell clones (40-44). A similar result was obtained by amino acid sequence analysis of the deposited amyloid combined with mRNA extraction and DNA sequencing (33). Therefore it has been suggested that the process can be classified as a ‘low-grade B cell lymphoproliferative disease' (44), a somewhat diffuse designation but one which in hematological terminology probably means a neoplasm.

### Pathogenesis of the amyloidogenic plasma cell clone

Given that the most common localization of localized AL amyloidosis is at mucous membranes in contact with the environment, it has been suggested there is an antigenic induction of amyloidogenic plasma cell clones (17). A chronic inflammation has often been encountered (15). It seems likely that similar plasma cell clones without amyloid deposits may appear as commonly when the cells do not produce an amyloidogenic Ig light chain. It is noteworthy that a number of cases with localized AL amyloidosis have been associated with Sjögren's syndrome (24,45).

### Giant cells in localized AL amyloidosis

**Figure 4.**
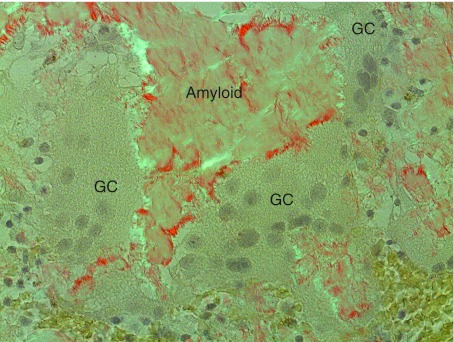
Amyloid adjacent to giant cells (GC). An organization of amyloid fibrils is apparent when in contact with giant cells. This organization is interpreted as assemblage of protein, probably after some modification, into fibrils on or at the surface of the cells. Congo red stained section in polarized light with crossed polars.

Foreign giant cells are very often mentioned in plentiful case reports on localized amyloidosis (e.g. (17,18,22,36,39,44,46-55)). Their occurrence is usually explained as a reaction to the amyloid deposits (54). However, amyloid in general does not induce a foreign body reaction, and there are almost never any giant cells present in the amyloid masses in systemic AL amyloidosis. Furthermore, amyloid fibrils close to giant cells are organized in the same way as seen at sites where cells participate in amyloid production, e.g. in the spleen in AA amyloidosis (56) or in the islets of Langerhans in IAPP amyloidosis (14). This is often suggested already when using polarization microscopy where a clear-cut orientation of amyloid fibril bundles against giant cells is evident (Figure 4). Therefore, we proposed that giant cells in localized AL amyloidosis directly participate in the transformation of the soluble full-length light chains into insoluble fibrils formed from N-terminal light chain fragments (34).

The genesis and function of multinuclear giant cells, typical of certain infections and inflammations, are still poorly understood. They develop by fusion of macrophages and are generally thought of as cellular elements that try to perform phagocytosis of particular structures, e.g. larger foreign particles. However, this simplistic explanation has been questioned, and giant cells may exhibit novel functions that are not yet understood (57). Experimentally, giant cells have been reported to be induced by cultivation of monocytes in the presence of interleukin 3 (IL-3) and interferon-gamma (58,59), IL-4 (60-64), or IL-13 (65). Plasma cells can express IL-4 (66). Although T cells may be a more important source of IL-4, there is a normal foreign body response in T cell-deficient mice (67). The inducer in localized AL amyloid is unknown, and giant cells do not appear in the bone-marrow in systemic AL amyloidosis in spite of presence of neoplastic plasma cells. It should also be pointed out that the bone-marrow is usually not particularly affected in the systemic disease, although the fibril protein is synthesized there.

## Hypothesis and conclusion

**Figure 5.**
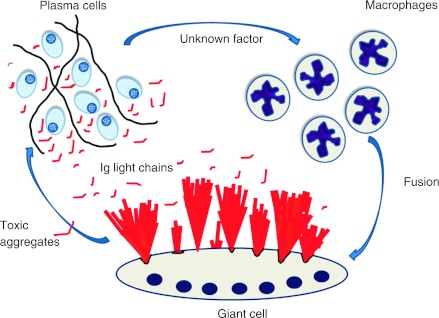
Suggested events in localized AL amyloidosis. A plasma cell clone develops at one site. The cells express amyloidogenic Ig light chains and, in addition, an unknown factor that attracts macrophages and cause them to fuse into giant cells. Ig light chains are modified by giant cells and aggregate into bundles of amyloid fibrils at the surface of these cells. In addition, smaller aggregates of Ig light chains are toxic to plasma cells, an event controlling the plasma cell clone.

From the current knowledge, it is only possible to create a hypothesis for the pathogenesis of localized AL amyloidosis. A benign small plasma cell clone is formed at one site, perhaps after prolonged antigenic stimulation. The clonal plasma cells produce an immunoglobulin light chain which is amyloidogenic but needs to be modified. Only clones that can recruit macrophages, which fuse to giant cells by mechanisms yet to be elucidated, can form amyloid. The modification of the light chains may take place within giant cells which take up the soluble light chain molecules by endocytosis and process them so that the N-terminal parts can aggregate into fibrils. Alternatively, protein modification may occur on the cell surface. Most likely, the assembly take place on the cell surface, e.g. after lysosomal secretion, which is a regulated secretory pathway (68). Such a scenario would explain the evident organization of amyloid fibrils close to giant cells, very different from what is seen in deposits in systemic amyloidosis. Another fact which points to a pathogenesis different from systemic amyloidosis is that localized AL amyloid of kappa type is common. Although it is only possible to speculate, this may depend on a lower propensity of kappa chains to form beta-sheet fibrils, overcome in the localized situation where the protein concentration close to plasma cells likely is high. The hypothetical formation of localized AL amyloidosis has been outlined in Figure 5.

Another interesting aspect of localized AL amyloid is the sparse occurrence of plasma cells in the lesions. In fact, the number of plasma cells is sometimes so low that the clonality can be difficult to demonstrate. The lesion has therefore been suggested to represent a ‘burnt-out' plasmacytoma (9,69). In many other amyloid diseases, e.g. Alzheimer's disease or type 2 diabetes, there is increasing evidence that amyloid protein aggregation involves genesis of smaller aggregates (oligomers, protofibrils) that exert a toxic effect on nearby cells and generate apoptosis (reviewed in (70,71)). In addition, such a mechanism is believed to explain the rapid effect on myocardial dysfunction after successful treatment of a plasma cell clone in systemic AL amyloidosis (72). If generation of toxic oligomers is true also with the formation of localized AL amyloid, this may lead to apoptosis of plasma cells in an ‘amyloidoma'. This event may then be regarded as a suicide of tumor plasma cells creating a self-limiting neoplasm.
